# Antioxidant and Antidiabetic Properties of Phlorotannins from *Ascophyllum nodosum* Seaweed Extracts

**DOI:** 10.3390/molecules28134937

**Published:** 2023-06-23

**Authors:** Mauro Gisbert, Daniel Franco, Jorge Sineiro, Ramón Moreira

**Affiliations:** 1 Chemical Engineering Department, Universidade de Santiago de Compostela, Campus Vida, 15782 Santiago de Compostela, Spain; mauro.gisbert@ucd.ie (M.G.); daniel.franco.ruiz@usc.es (D.F.); jorge.sineiro@usc.es (J.S.); 2 School of Mechanical and Materials Engineering, University College Dublin, Stillorgan Rd, Belfield, Dublin 4, D04 V1W8 Dublin, Ireland

**Keywords:** ultrasound-assisted extraction, phlorotannins, solid–liquid extraction, antioxidant activity, functional food, α-amylase, α-glucosidase, polyphenols, bioactivity, digestive enzymes inhibition

## Abstract

Seaweeds have gained considerable attention in recent years due to their potential health benefits and high contents of bioactive compounds. This review focuses on the exploration of seaweed’s health-promoting properties, with particular emphasis on phlorotannins, a class of bioactive compounds known for their antioxidant and antidiabetic properties. Various novel and ecofriendly extraction methods, including solid–liquid extraction, ultrasound-assisted extraction, and microwave-assisted extraction are examined for their effectiveness in isolating phlorotannins. The chemical structure and isolation of phlorotannins are discussed, along with methods for their characterization, such as spectrophotometry, nuclear magnetic resonance, Fourier transform infrared spectroscopy, and chromatography. Special attention is given to the antioxidant activity of phlorotannins. The inhibitory capacities of polyphenols, specifically phlorotannins from *Ascophyllum nodosum* against digestive enzymes, such as α-amylase and α-glucosidase, are explored. The results suggest that polyphenols from *Ascophyllum nodosum* seaweed hold significant potential as enzyme inhibitors, although the inhibitory activity may vary depending on the extraction conditions and the specific enzyme involved. In conclusion, seaweed exhibits great potential as a functional food ingredient for promoting health and preventing chronic diseases. Overall, this review aims to condense a comprehensive collection of high-yield, low-cost, and ecofriendly extraction methods for obtaining phlorotannins with remarkable antioxidant and antidiabetic capacities.

## 1. Introduction: The Importance of Macroalgae in the Food Industry

**Figure 1:**
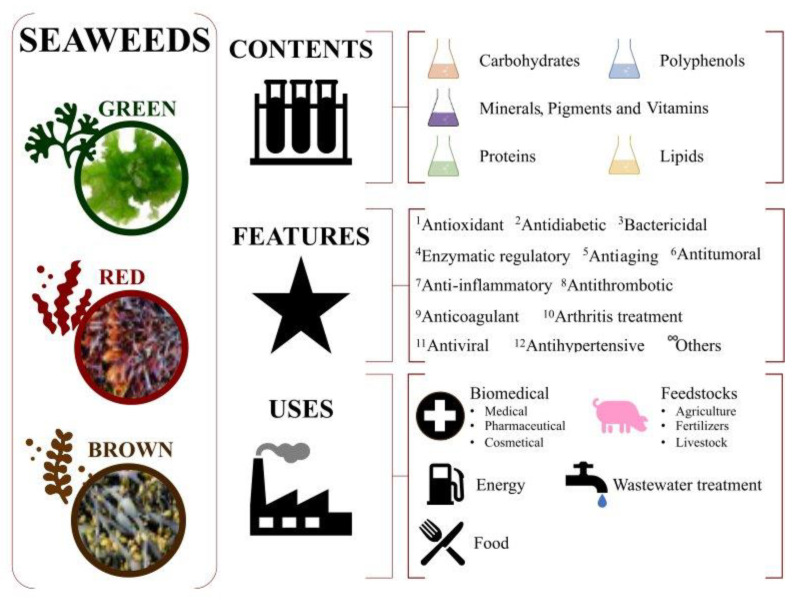
Potential uses of seaweeds. Market and applications.

Macroalgae, commonly known as seaweeds, are gaining attention due to their rich contents of bioactive compounds (e.g., 2-phloroeckol, 6,6′-bieckol, 7-phloroeckol, eckol, fucophlorethols, fucodiphloroethol G, phlorofucofuroeckol A and B, tetraphlorethols E, and/or triphlorethol). They have emerged as an interesting and sought-after resource in the field of biotechnology, with their relevant health benefits exploited by several industrial sectors such as the biomedical [1], feedstock [2], biofuel production [3], wastewater treatment [4], and food [5] industries, as summarized in Figure 1.

This rising global consumption of seaweeds and their derived products is generating notorious demand with socioeconomic relevance, which may increase revenue through the processing of high-value products by up to USD 26 million by 2025 [6]. According to FAO [6], this trend will increase by up to 10% in the coming years. For instance, the number of cosmetic products that include seaweed compounds is increasing. It is common to see product labels containing terms such as “marine extract”, “algae extract of algae”, “seaweed extract”, or similar. For example, alginate increases the skin moisture retention properties of some lotions [7]. Seaweed-derived pastes are commonly used in thalassotherapy together with hydrotherapy to partially relieve rheumatism and osteoporosis problems [8]. Regarding energy production, seaweeds are also being explored as a sustainable source of biofuels. They are a potential alternative to fossil fuels due to their high growth rate, low land and water requirements, and ability to absorb carbon dioxide [3]. The direct combustion of algae biomass is a traditional method of generating heat or steam, but it is not a suitable method for energy production due to producing emissions together with its low efficiency. On the contrary, the production of biofuel from seaweeds can be carried out in the presence of catalysts and hydrogen at high pressure and lower temperatures via a hydrothermal liquefaction process [9]. In wastewater treatment, some compounds from seaweeds were demonstrated to be efficient chelators to remove hazardous pollutants such as heavy metals from industrial down-streaming [10]. Regarding their use as fertilizers, it was demonstrated that seaweeds enhance soil moisture retention, and their mineral content is a source of trace essential elements [11]. Additionally, seaweeds have been included in commercial feeds for cattle, having positive health benefits for livestock, such as reducing the need for antibiotics in pig rearing [12].

The food industry is the largest consumer of seaweeds. The use of seaweeds in the food industry as food has strong roots in Asian countries such as China, Japan, and Korea, being less consumed in Western countries [13]. Brown seaweed extracts are being studied for use as food additives [14]; to replace chemical preservatives because of their antioxidant and antimicrobial activities [15]; and to open new prospects in the elaboration of novel, attractive, and healthy foods. More specifically, the benefits of macroalgae as a source of novel bioactive products are being revealed by the scientific community with the increasing interest in their different biological activities, and consumers are more attracted to marine-algae-derived foods [16,17]. Seaweeds are a rich source of minerals, vitamins, and dietary fiber and have a low caloric content, making them an ideal ingredient for functional foods [5]. Seaweeds compounds are also used as thickening agents, stabilizers, and emulsifiers in food processing and provide promising and important compounds, such as bioactive phloroglucinol-derived structure phenolic compounds, unsaturated fatty acids, fucoidans, alginate, and biopolymers [13]. Currently, there are no known limits or possible related harmful effects on either animal or human health with respect to the consumption of seaweeds, except those related to the high iodine levels and, consequently, the derived recommendation of avoiding excessive frequent ingestion. For centuries, seaweeds have generally been considered safe to consume in relatively large quantities, especially in oriental countries. Metals, including lead, arsenic, cadmium, and mercury, can naturally accumulate in seaweeds due to a variety of factors such as environmental contamination, industrial activities, or uptake from seawater. These metals can pose significant health risks when consumed in excessive amounts, potentially leading to adverse effects on the nervous system, kidneys, liver, and other vital organs, limiting their applications as feedstock (human and animal) [18]. Nowadays, seaweeds are considered a “novel food” under European regulations (Regulation (EU) 2017/2470). The development of robust quality control measures and standardized protocols for metal analysis and purification processes is crucial to ensure consistency and reliability when assessing and mitigating metal contamination in seaweeds. Regulatory agencies worldwide have often established maximum permissible limits for metals in food products, and strict adherence to these regulations is paramount in guaranteeing the safety and well-being of consumers.

**Figure 2:**
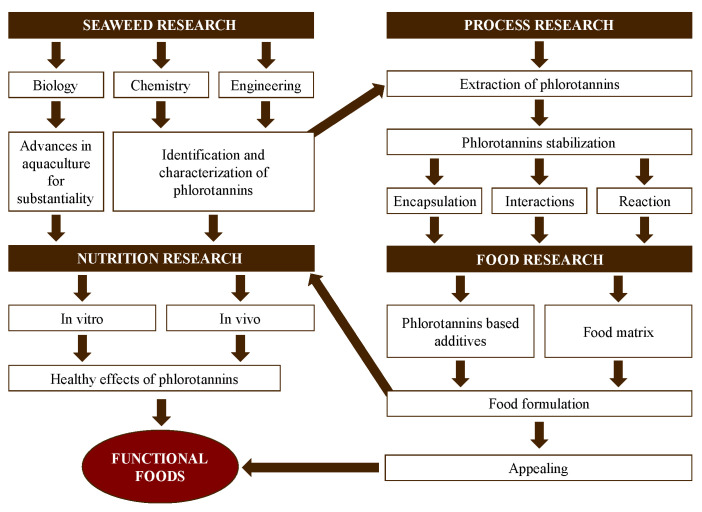
Stages in the development of functional foods using seaweed as a source of phlorotannins from a multidisciplinary point of view.

In the past decades, the interest in and research regarding functional foods have considerably increased. Functional foods contain bioactive compounds with known health benefits beyond the role of basic nutrition [19]. Antioxidant capacity is essential to ensure the quality of functional foods and prevent and treat diseases related to oxidative stress [20]. The development and validation of functional foods is, in general, a slow and expensive process that includes the chemical characterization of natural sources, extraction, optimization of purification methods and sequences, food designing, and, finally, in vivo validation [21]. The use of seaweeds as natural sources of new functional foods is a promising field. This should be approached via integrated and complex approaches, considering different research areas (seaweeds, processing, food product, and nutrition) with multiple interactions among them (Figure 2). Additionally, the use of seaweed as a food supplement for the control of the glycemic index has been investigated [22,23] and was explored in this review along with its antioxidant activity.

For the brown seaweeds used as a source of bioactive compounds, it is essential to increase the number of biological studies where new edible species are identified and characterized. The main goal is to increase current knowledge to achieve a suitable design of functional foods using seaweed as a source of bioactive ingredients. This review also partly focused on the production of some of these ingredients (phlorotannins and alginates) extracted from *Ascophyllum nodosum* (*A. nodosum*) seaweeds.

## 2. Phlorotannins

**Figure 3:**
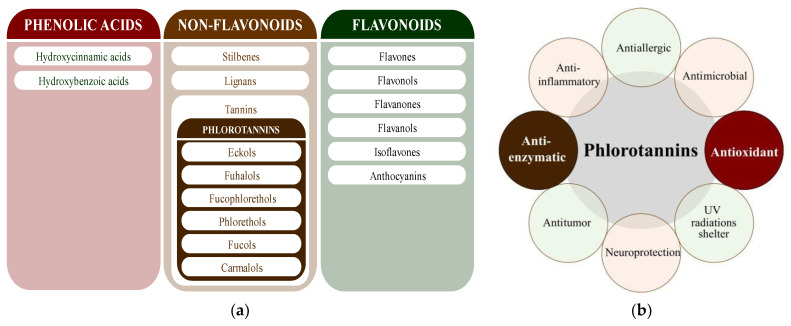
(**a**) Main three chemical families of polyphenols; (**b**) Main biological activities of phlorotannin’s.

Polyphenols are secondary metabolites from terrestrial and marine plants and lichens and generally act as structural cell wall components and protection against environmental stress. They are aromatic compounds with more than one hydroxylic group and can be divided into three categories: phenolic acids, flavonoids, and no-flavonoids [24,25], as depicted in Figure 3a. Among them, phlorotannins have received significant attention in the fields of research, industry, and medicine due to their bioactive activities such as antioxidant, anticoagulant, antithrombotic, antidiabetic, enzymatic regulatory, antimicrobial, antitumoral, anti-inflammatory, antihypertensive, and antiviral activities, among others [15,26,27,28] (Figure 3b). In addition, they have shown promising results in the treatment of Alzheimer’s and Parkinson’s disease and arthritis [27,29]. 

**Figure 4:**
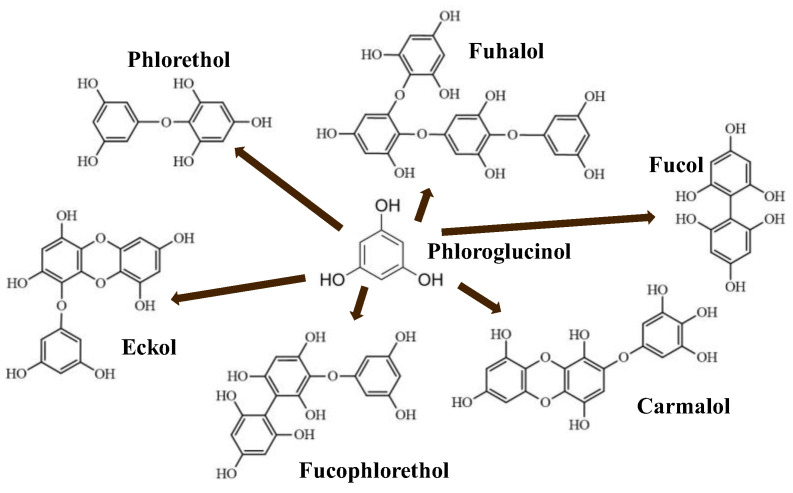
Phlorotannin main groups derived from phloroglucinol polyketide pathway reaction.

Phlorotannins are moderately hydrophilic components with a wide range of molecular weights, ranging between 126 and 650 kDa. They are produced via the polymerization of the phloroglucinol molecule (benzene-1,3,5-triol) through the polyketide pathway reaction and stored in physodes and/or cell-wall-forming complexes. The content of phlorotannins in seaweeds depends on environmental conditions, such as tides, salinity, light availability, UV radiation, and herbivory intensity. These compounds are divided into six different classes according to the variations in their assemblage and distribution of hydroxyl groups: eckols, fuhalols, fucophlorethols, phlorethols, fucols, and carmalols (Figure 4). Phlorethols and fuhalols present aryl–ether linkages, fucols aryl–aryl bonds, fucophlorethols, and a mixture of ether and phenyl bonds; eckols present a 1,4-dibenzodioxin unit. Carmalols present a 4-dibenzodioxin unit at the third and seventh positions. Eckols differ from carmalols in their lower molecular weight and the presence of an OH group substituted at the fourth carbon; fuhalols differ from phlorethols in their regular sequence of para- and ortho-ether bonds, the presence of additional OH groups in every third ring, and the lack of one or more OH groups in the whole molecule [13,30,31].

## 3. Phlorotannins’ Extraction and Isolation

**Figure 5:**
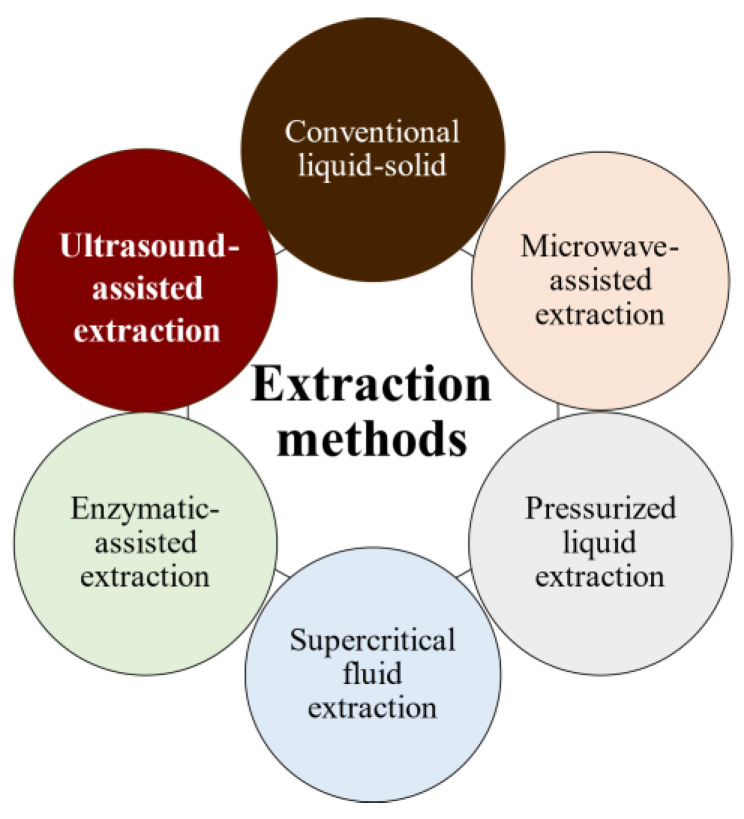
Phlorotannins’ extraction methods.

The bioactivities and characteristics of phlorotannins as well as their amount are influenced by the extraction method used and the employed conditions (e.g., operation mode, solvent, solid–liquid ratio, time, temperature, and pre- and post-treatments) [32,33]. For instance, the selection of the operation mode is a key aspect of industrial production. Batch extraction requires interruptions for charging, discharging, and cleaning steps and high amounts of solvent. These problems are reduced under semicontinuous extraction consisting of several batch extractors operated in series. Another important factor is the solvent used for the extraction to achieve high operation yields and minimize the coextraction of undesirable substances. Indeed, the extraction of phlorotannins is a challenge because they are deeply embedded among the seaweed components, forming complexes [34]. In addition to the solvent, phlorotannins’ solubility is influenced by the polymerization degree and interactions with other food constituents [35,36]. Despite organic solvents being largely recommended for the extraction of antioxidants from plants and seaweeds in terms of extraction yield, these solvents are volatile, inflammable, and/or toxic [37,38,39]. As an alternative, water is being proposed as an efficient and green-labeled alternative for polyphenol extraction from seaweed [32,40,41]. Here, several extraction methods have been tested to extract bioactive compounds from algal material, aiming to develop new, safe, effective, and affordable extraction technologies to minimize the presence of residues. Moreover, in recent years, there has been growing interest in developing greener and cleaner extraction technologies. These methods (Figure 5) are being extensively studied to ensure that they are not only effective but also environmentally friendly and sustainable [31,42,43]. 

Pressurized liquid extraction (PLE) utilizes high pressures (up to 15 MPa) and temperatures (up to 200 °C), along with low extractant volumes and short extraction times, whereas microwave-assisted extraction (MAE) uses electromagnetic waves to induce and facilitate compound extraction. However, both methods promote the partial degradation of thermolabile compounds [43]. Supercritical fluid extraction uses fluids with a temperature and pressure above their critical point (most often CO_2_) to increase mass transfer by decreasing surface tension and viscosity, but it is less used because of the high costs of equipment and solvent (if no solvent is recovered); additionally, for optimal results, the solvent polarity must be tuned by adding polar compounds (alcohols) to CO_2_ for phenolics extraction [44]. 

During phlorotannins extraction, other biomolecules are coextracted, mainly polysaccharides and proteins; hence, the separation and purification for the fractionation and/or isolation of desired compounds are recommended. Extract fractionation consists of separation based on molecular weights, charges, chemical affinities, and/or solubilities [28,45]. Adsorption-based separation methods, such as flash chromatography, are emerging among the fractionation techniques due to their simplicity, the potential for scale-up, and higher specificity compared with those of other primary fractionation techniques [45]. Separation is achieved by the contact of seaweed extracts with a solid matrix with different affinities for phlorotannins and the remaining compounds. Then, phlorotannins can be recovered by separating the solid and liquid phases. In addition to solid-phase extraction, in which the sorbent is immobilized on a cartridge or column, allowing sequential elution of compounds with a solvent gradient, liquid–liquid extraction is a solubility-based separation method in which the wide range of polarities of phenolic compounds allows their relatively easy partitioning. Another possibility is based on ultrafiltration and/or molecular-weight cut-off dialysis. These techniques require minimal instrumentation and expertise and allow the quick separation of fractions over a wide range of molecular weights using only a few combinations of membranes or filters. Two extraction methods have been extensively studied for extracting phlorotannins: conventional solid–liquid extraction (SLE) and ultrasound-assisted extraction (UAE). In this review, we focus on these two methods.

### 3.1. Solid–Liquid Extraction

**Figure 6:**
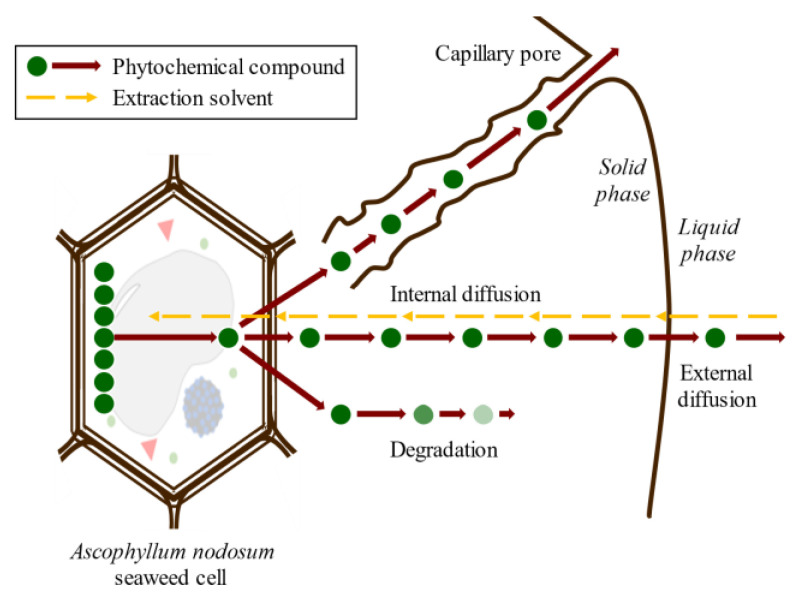
Scheme of the simultaneous stages of SLE of compounds from seaweed cell.

Conventional SLE, also known as leaching, is a widely used method in the food industry to extract various compounds such as sucrose, lipids, proteins, phenolic compounds, and hydrocolloids [32,46,47]. Molecular diffusion is the primary transport mechanism in this process, where compounds are transported by a concentration gradient in the solid phase. The microstructure of the solids plays a significant role in the rate and characteristics of the extraction process due to factors such as porosity, pore size, and moisture content. The microstructure refers to the arrangement and characteristics of the solid material at the microscopic level, including the distribution and size of pores or void spaces within the material. SLE is a complex process involving multicomponent, multiphase, and unsteady-state mass transfer operations, which are affected by the relative migration rate of different compounds through the solid. The SLE process can be divided into several stages that can occur simultaneously or, in some cases, sequentially: (1) solvent entrance into a solid matrix; (2) solubilization/breakdown of components; (3) solute transport to the solid matrix surface; (4) extracted solute migration from the solid surface through the solvent bulk; (5) separation of the extract and solid. Understanding each of these stages is crucial for optimizing the SLE process and improving the extraction yield. Compound transport through the solid matrix is usually the rate-controlling step, resulting in an energy- and time-consumption process that requires potentially high amounts of solvents to produce acceptable extraction yields [48]. The extraction of bioactive compounds at a commercial scale requires high extraction yields and the conservation of their bioactivities, which are difficult to achieve using the conventional SLE method [49]. The simultaneous processes involved in the SLE of compounds from seaweed cells are shown in Figure 6.

### 3.2. Ultrasound-Assisted Extraction 

**Figure 7:**
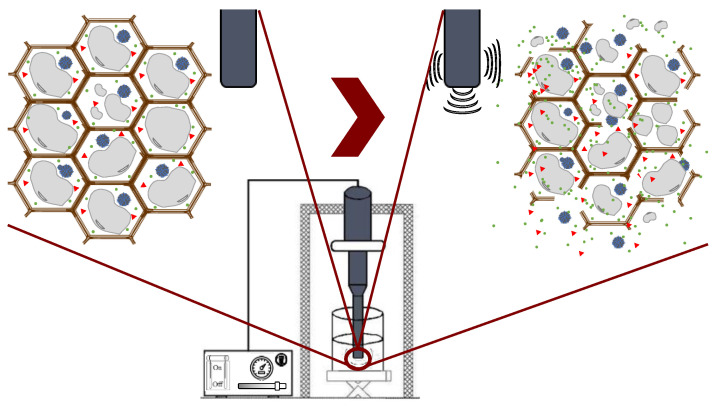
Ultrasound-assisted extraction process.

Ultrasound technology is widely used in the food industry because it can be used either as a pretreatment or can be combined with different types of solvents [25,50]. Ultrasound comprises mechanical waves, ranging from 20 kHz up to 10 MHz, involving various phenomena such as shear forces, compression pressure gradients, agitation, rarefaction, vibration, microjets, radical formation, and cavitation [42]. UAE utilizes soundwaves to disintegrate the cell structure for the subsequent release of compounds. Cavitation is the main force driving ultrasound extraction: it is a hydrodynamic effect that occurs when vapor cavities are created within the liquid, and different pressure forces are present [51]. These processes produce the expansion and implosive collapse of microbubbles, formed via a series of compressions and rarefactions in molecules generated by ultrasound waves, improving heat and mass transfer along the system and improving solvent penetration and cell-wall breaking. The main advantages of UAE are its reduced solvent consumption, shorter extraction time, lower operational costs, minimal impact on the stability of the target compounds because high temperatures are not required, and higher process efficiency and extraction yields compared with conventional extraction methods [42,52]. Figure 7 shows the general scheme of ultrasound-assisted extraction from seaweed tissues.

In the last twenty years, several researchers have studied SLE and UAE features, such as solvent type, liquid–solid ratio, time, and temperature as critical aspects of phlorotannin extraction from *Ascophyllum nodosum* brown seaweeds. However, there are limited data on the effect of using UAE to produce phenolic-compound-enriched extracts [53]. Phlorotannins are measured as total polyphenol content (TPC) based on the Folin–Ciocalteau method (see Section 4.1.1). Additionally, different standards have been used for quantification of, for example, gallic acid, phloroglucinol, pyrocatechol, raw phlorotannin, and catechin. The establishment of a standardized protocol to quantify algal antioxidant extract activity would be convenient. The main studies are summarized in Table 1. According to the reviewed literature, the TPC values have ranged from 0.7 mgPE/mg extract [46] to 0.5 g PE/g extract [46], working with ethanol at 96% and 40% *v*/*v*, respectively. Furthermore, the lowest TPC values were obtained using SLE, with a high liquid–solid ratio (LS = 90 g solvent/g d.b) and an intermediate operational time (t = 30 min) and temperature (T = 30 °C). On the contrary, the highest extraction was achieved using UAE, with a lower LS value (50 g solvent/g dry basis (d.b)), similar time (30 min), and higher temperature (60 °C). According to the literature, organic solvents have been commonly used for extracting phlorotannins, the extraction procedures have shown increased extraction yields, and low temperatures have typically been used to prevent the thermal degradation of phytochemicals.

While many chromatographic techniques have been utilized for separating, isolating, purifying, identifying, and quantifying individual phenolic compounds from plant materials, research on individual phenolic compounds in brown algae remains limited. To enhance our understanding of the bioactive potential of brown-algae-derived phenolic compounds, we need to know the chemical and physical structure. Table 2 shows some of these studies regarding the phlorotannins isolated from brown seaweeds. The isolation of phlorotannins from different seaweed involves many steps, large solvent amounts, a long time, and large amounts of energy, making the process complicated and expensive. This explains the scarcity of standards and the current lack of commercial availability of phlorotannins. Nevertheless, the isolation of these compounds is required to understand their bioactivity for further use in real applications.

## 4. Characterization of Seaweed Phlorotannins

**Figure 8:**
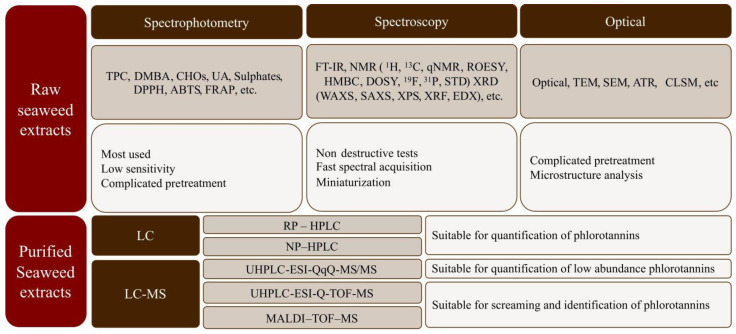
Summary of analytical methods most often used for characterization of seaweed biopolymers.

The chemical characterization of both crude and purified compounds from seaweeds is relevant in food engineering, medicine, and pharmacy to understand their bioactivities and beneficial health properties [20]. Nevertheless, isolation is often difficult due to their diverse molecular weights, structural similarities, and rapid reactivities [45,82]. Seaweed extracts are composed of a large, diverse, and complex mixture of compounds, where the phlorotannins are found together with polysaccharides, proteins, and other compounds. Initially, raw characterization is commonly carried out via spectrophotometric assays that are simple, cheap, and rapid, which enables comparison among studies [20,83]. Additionally, nuclear magnetic resonance (NMR), Fourier transform infrared spectroscopy (FT-IR), Matrix-assisted laser desorption ionization-time of flight mass spectrometry (MALDI-TOF-MS), and chromatographic techniques (HPLC and/or UPLC) have been reported as more reliable techniques for seaweed extract characterization because they allow qualitative and quantitative estimation [83]. Figure 8 shows a summary of the analytical methods often used for the characterization of seaweed biopolymers.

In the following sections, a comprehensive overview of the characterization techniques suitable for application to seaweed extracts and phlorotannins, with special emphasis on antioxidant activity, is provided. This knowledge of analytical methods available improves the understanding of bioactive compounds in seaweed and their potential uses.

### 4.1. Antioxidant Activity Determination: Spectrophotometry Methods

**Figure 9:**
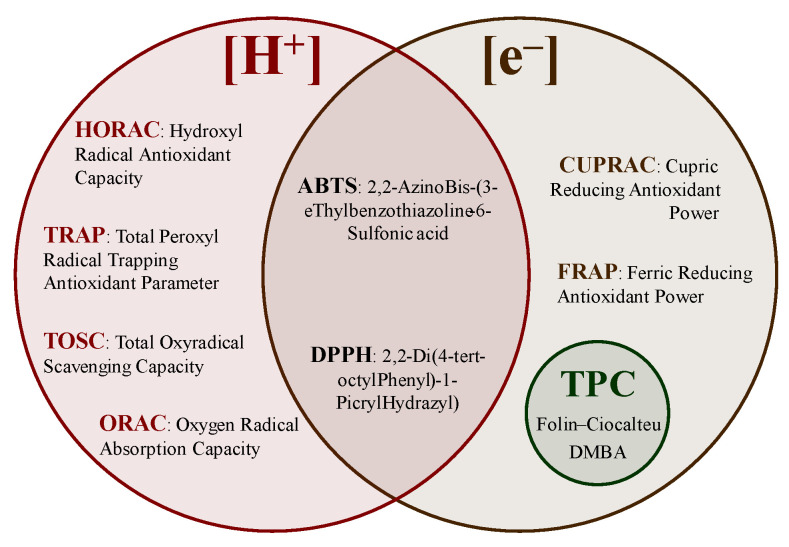
Antioxidant activity determination methods.

The background of the methods used to understand the reaction mechanisms of antioxidants and the advantages and limitations of the different tests are described in this section. Spectrophotometric methods rely on the linear relationship between absorbance and concentration using the coloration/discoloration of a solution measured at a specific wavelength [13]. Currently, the available methods can be divided based on the transfer of hydrogen atoms ([H^+^]), electrons ([e^−^]), and/or a mixture of both (Figure 9), such as hydroxyl radical antioxidant capacity (HORAC), total radical-trapping antioxidant parameter (TRAP), total oxyradical scavenging capacity (TOSC), oxygen radical absorption capacity (ORAC), 2,20-azinobis-(3-thylbenzothiazoline-6-sulfonic) acid (ABTS), 2,2-di(4-tert-octylphenyl)-1-picryl hydrazyl radical) (DPPH), cupric-reducing antioxidant power (CUPRAC), Ferric-reducing antioxidant power (FRAP), and the TPC determinations [20,84,85,86,87].

#### 4.1.1. Determination of Total Polyphenols Content (TPC)

The total polyphenols content (TPC) test, based on the Folin–Ciocalteu method, is the most common assay used with (terrestrial and marine) polyphenol-enriched products. It was originally reported by Singleton and Rossi [88] as a method to analyze the phenolic components in red wine, becoming a routine test for the antioxidant evaluation of food and plant extracts, although it has the disadvantage of interfering with proteins [89]. The TPC test detects the compounds suitable to transfer electrons from reductant compounds to the molybdenum complexes of the Folin–Ciocalteu reagent, which promotes a color change, detected at 765 nm. The results are commonly expressed in gallic acid or catechin equivalents for plant extracts. For brown algae extracts, the use of calibration curves with phloroglucinol standard is recommended. 

#### 4.1.2. Antioxidant Methods Based on the Transfer of Hydrogen Atoms

Oxygen radical absorbance capacity (ORAC): This test is used to determine scavenging capacity through the inhibition of the oxidation of peroxyl radicals, which predominate in lipid oxidation in biological systems [87]. This test is based on the reaction of peroxyl radicals from a generator (e.g., azo compounds) that reacts with a fluorescent (e.g., fluorescein) sample, leading to the loss of fluorescence due to the antioxidant effect [90].

Hydroxyl radical absorbance capacity (HORAC): This method measures the ability of antioxidants to avoid the complexation reaction between hydroxyl radical complexation and cobalt ions (Co^2+^). Fluorescein, as a fluorescence source, is incubated with the assayed antioxidant samples, and then a generator of hydroxyl radicals is added (e.g., the Fenton mixture). The decay of fluorescence provides a direct measurement of antioxidant capacity [20,91].

Total radical-trapping antioxidant parameter (TRAP): This method measures the assayed compound’s capacity to inhibit the reaction between peroxyl radicals and target molecules using the oxygen consumption during the peroxidation process of 2,20 azo-bis(2-amidinopropane) dihydrochloride. The induction time for oxygen absorption is used to determine the total antioxidant capacity of the samples [86].

Total oxyradical scavenging capacity (TOSC): This test is based on the inhibition of the formation of ethylene in the presence of antioxidant compounds that compete with α-keto-γ-methiolbutyric acid (KMBA) for oxidants. This test uses the relationship between the area under the ethylene concentration curve, obtained via gas chromatography, and the reaction time between KMBA and oxidants [92].

#### 4.1.3. Antioxidant Methods Based on Electron Transfer 

Cupric-reducing antioxidant capacity (CUPRAC): This assay determines the total antioxidant capacity based on the reduction of cupper ions (Cu^2+^ and Cu^+^) within the reaction mechanism of the ligand with neocuproine (2,9-dimethyl-1,10-phenanthroline), whose color is determined at 450 nm, indicating the antioxidant activity of the samples [20].

Ferric-reducing antioxidant power (FRAP): The FRAP test is used on a large scale with foods [85]. The method is based on the reduction of iron (Fe^3+^ to Fe^2+^) as a ligand via the effect of antioxidant compounds. Antioxidant activity is determined as an increased color measured at 593 nm [93].

#### 4.1.4. Antioxidant Methods Based on Proton and Electron Transfer

DPPH method: 2,2-diphenyl-1-picrylhydrazyl (DPPH) is a stable radical that is soluble in different organic solvents but not in water [94]. The DPPH test is based on the DPPH radical scavenging of the electrons donated by the assayed antioxidant [95]. This reaction produces a discoloration measured at 515 nm, indicating the scavenging activity decay of DPPH^●^. It is usually reported as IC_50_, (i.e., the concentration of the antioxidant necessary to reduce the initial DPPH concentration up to 50%). This test is widely used due to its low cost, ease of use, reproducibility, and ability to operate at room temperature [20]. 

ABTS method: This method is based on the reaction with 2,2′-azinobis-(3-ethylbenzothiazoline-6-sulfonic acid (ABTS^+^), which is a stable radical with radical scavenging molecules, and expressed as Trolox equivalent antioxidant content (TEAC). This test measures the antioxidant’s capacity to neutralize ABTS^●^ radicals using a discoloration method at 734 nm [96]. The ABTS assay can be used over a wide pH range; the radical is soluble in both water and organic solvents, which allows the determination of the antioxidant capacity of both hydrophilic and lipophilic compounds [97,98].

### 4.2. Chromatographic Methods

High-performance liquid chromatography (HPLC) is widely used to identify the structure and linkages’ positions, type, and size of phlorotannins and quantify them [13,38]. Normal-phase HPLC (NP-HPLC) uses a polar stationary phase to separate compounds based on their polarity, but the strong interactions of polar phlorotannins with the stationary phase can make their elution difficult. For this reason, reverse-phase HPLC (RP-HPLC) is commonly used for phlorotannin analysis due to its better reproducibility and lower retention times [99]. Another possibility is the use of ultra-performance liquid chromatography (UPLC), which reduces solvent usage and column size and increases the speed and sensitivity of the analysis [100]. Additionally, size exclusion chromatography (SEC) has been used for the preparative separation of different molecular-weight fractions from seaweed extract as well as to confirm the molecular size of an isolated metabolite [67].

Regarding identification, phlorotannins typically absorb ultraviolet radiation in the range of 260 to 330 nm. Thus, detectors based on visible and UV–Vis are the simplest and most widely applicable to this kind of molecule [13]. However, the absorption of phenolic compounds can significantly vary due to the similarity of their structures, factors such as pH, as well as the presence of other components. As a result, peak identification using UV–Vis detectors can be ambiguous, particularly in the case of closely related compounds such as phlorotannins. A possible improvement would be to use a diode array detector (DAD) for the analysis of compounds with similar molecular weights but different electronic distributions in the chromophore, which leads to different UV spectra. Indeed, HPLC combined with a UV–Vis or a DAD detector and C18 column is the main system used for the separation and/or quantification of phlorotannins [101,102]. Alternatively, the use of mass spectrometers (MS)-based on the measuring mass–charge ratio (*m*/*z*) directly coupled to U/HPLC instruments minimizes this problem by increasing the capacity to analyze complex extracts, allowing the qualitative and quantitative of hundreds of polyphenolic components. Moreover, MS detection can easily provide a profile of the degree of polymerization in phlorotannins-enriched extracts [36,38,54,103]. Among the several MS fragmentation methods, electrospray ionization (ESI) is the most-often-used ionization procedure for phlorotannins in U/HPLC systems. These compounds are ionized in negative-ion mode to produce deprotonated molecular ions; sometimes, in seaweed extracts from complex matrices, interference from coeluting compounds can occur, leading to ionization suppression or enhancement in the signals [104]. In those cases, purification is required during sample preparation via LC-MS, increasing procedure time and cost [102]. The isomerization by multiple combinations of phloroglucinol units after ionization has a strong impact on mass spectra; often, it is difficult to attribute a chemical structure to the detected molecule. Overall, the main limitation of ESI is that the sample is vaporized, which does not permit the analysis of higher-molecular-weight and thermally labile components [76]. To overcome these problems, matrix-assisted laser desorption ionization (MALDI) combined with a time of flight (TOF) analyzer is a particularly suitable technique for the analysis of larger oligomers with *m*/*z* values above the upper limit of ESI-MS. Indeed, this chromatographic technique has also been used in combination with U/HPLC-ESI-MS to provide information about the size of and isomeric variation in phlorotannins [105]. Although the identification of phenolic compounds on macroalgae has increased in the last few years, there is still a lack of information on the structural identification of higher-molecular-weight compounds, particularly in the case of phlorotannins. The methods used to fractionate these extracts and obtain phenolic-compound-enriched fractions are still insufficient in preparing the extracts for analytical platforms. It is still necessary to reduce the complexity of extract matrices for analysis purposes. Therefore, it is of crucial importance to develop novel extraction and primary fractionation methodologies that enable the identification of phlorotannins. 

### 4.3. Fourier Transform Infrared Spectroscopy (FT-IR)

Infrared spectroscopy provides information to identify materials, their composition, and their functional groups (proximate molecular structure) and can monitor the course of a reaction based on the information on the relative vibrations among the atoms [106]. The absorption of infrared light by molecules is recorded in the infrared region (12,800 to 50 cm^−1^, with the range 4000–600 cm^−1^ being the most useful for the qualitative analysis of organic molecules) to obtain the infrared spectrum. It is a nondestructive and fast acquisition technique in which chemical reagents are not necessary. The FT-IR has been a widely used method in the characterization of phenolic groups in macroalgae extracts via the simultaneous occurrence of bands of hydroxyl groups at 3000 to 3500 and aromatic rings at 1200 to 1700 and at 2850 to 3000 cm^−1^ [15,66,82,107].

### 4.4. Nuclear Magnetic Resonance (NMR)

The NMR technique provides information about the functional groups and placement of structural moieties in molecules. NMR spectroscopy is an adequate method for identifying phlorotannins and can be used to determine their purity [108]. NMR allows the analysis of complex mixtures, being suitable for use in raw extract analysis [82]. Because of the presence of many different hydroxyl groups, the NMR spectra of phlorotannins-enriched extracts can sometimes be complicated to interpret. Labile protons from OH generally exhibit broad signals that can hamper interpretation. A solvent, when samples are diluted and measured, is critical because its polarity may change the equilibrium of the interactions formed, altering peak size, distribution, and shape in the NMR spectrum. Deuterated water (D_2_O) is one of the most used in the NMR literature, but it requires the use of high temperatures [109]. For this reason, the analysis of seaweed extracts is recommended to be carried out using deuterated DMSO (DMSO-d_6_) [110]. The aromatic protons of phloroglucinol often range between 6.0 and 7.5 ppm [13,111]. Hydrogen (^1^H-) and carbon (^13^C-) NMR are the most frequently used for the analysis and/or identification of phlorotannins. However, several NMR techniques can be employed such as 2-dimensional NMR (2D-NMR), heteronuclear multiple-quantum correlation (HMQC), fluor (^19^F-NMR), phosphorus (^31^P-NMR), heteronuclear multiple-bond correlation (HMBC), quantitative NMR (qNMR), or ^1^H-^1^H correlation spectroscopy (COSY), among others [79].

### 4.5. X-ray Diffraction (XRD)

X-ray diffraction (XRD) has been used for the analysis of structures’ crystallinity in the biopolymers extracted from seaweeds [111,112]. Some variants of this technique are wide-angle X-ray scattering (WAXS) and small-angle X-ray scattering (SAXS), which complement XRD, providing additional information about the structure of materials at the nano- and mesoscale. X-ray photoelectron spectroscopy (XPS), electron spectroscopy for chemical analysis (ESCA), as well as secondary ion mass spectroscopy (SIMS) provide information about elemental composition, surface chemistry, and adhesion mechanisms in layers. X-ray fluorescence (XRF) may also provide information about the elemental composition of assayed samples [113].

### 4.6. Microscopy Techniques

Transmission electron microscopy (TEM) is an imaging technique used for analyzing the interior of materials at the atomic level with significantly higher (thousands of times) better resolution than light microscopes. Scanning electron microscopy (SEM) allows the observation and surface characterization of inorganic and organic materials, delivering morphological information on the analyzed material. It can be coupled with X-ray detectors, such as an Energy-dispersive spectrometers (ED) or wavelength dispersive spectrometer (WDS) to semiquantitatively analyze the elemental composition of samples [114]. Confocal laser scanning microscopy (CLSM) is used to illuminate the subsurface layer of a specimen. This technique can be applied for biopolymer characterization to localize phenolic compounds within seaweed tissues; however, very few papers have been published applying this technique to seaweed biopolymers [115]. The large depth of the light microscope produces images containing information from different focal planes in thick specimens; good-quality and high-resolution images of the internal structure of samples can only be obtained from smears, squashes, or thin sections of the samples. Procedures that apply substantial shear and compressive forces may destroy or damage structural elements, and sectioning is time-consuming and involves chemical processing steps that may introduce artefacts and make image interpretation difficult. Attenuated total reflectance (ATR) is used to analyze surfaces; it is mainly used for adhesion problems, weathering plastic parts, and polymer films but it can also be used, combined with IR, with organic solutes in water [109].

## 5. Potential Effects of Phlorotannins as Starch Digestive Enzymes Inhibitors in the Control of Glycemic Index

**Figure 10:**
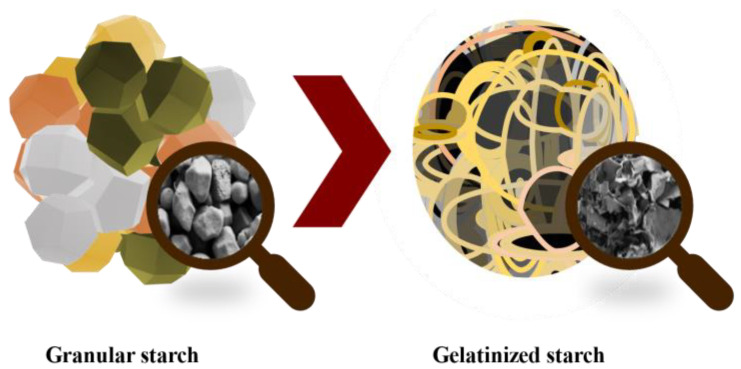
Starch gelatinization process and their microstructure changes.

The glycemic index (GI) is a measure of how quickly carbohydrates in food are broken down and absorbed by the body, which can affect blood sugar levels. Starch is the main contributor to the GI of many foods. Starch is formed by glucose molecules forming two types of bonds: α-1,4, which produce a linear structure called amylose; and α-1,6 bonds, which form branches called amylopectin. Starch varies in granule size, shape, and amylose/amylopectin ratio, depending on the plant’s origin [116]. These granules or native starches have a compact semicrystalline structure with low enzymatic accessibilities, which lead to low degradation rates and the low release of total sugars during digestion, making native starch a poor source of energy (Figure 10). When starch granules are heated in the presence of abundant water (e.g., during the cooking process), gelatinization takes place, and hydrogen bonds that stabilize the starch chains are broken, making amorphous starch more digestible [117]. After gelatinization, starch chains do not stay fully disordered and tend to partially reorder in a phenomenon called retrogradation, which depends on temperature, time, and molecule size/branching [116]. Overall, this recrystallization partially recovers the initial properties of starch [118].

Starch digestion takes place in the mouth and mainly in the small intestine through the action of several enzymes. Throughout the digestion of starch and other carbohydrates, α-amylase and α-glucosidase are the two key enzymes involved. α-amylase is secreted by the salivary glands and the pancreas, and it catalyzes the hydrolysis of the α-D-1,4 glycosidic linkages of starch, generating shorter oligosaccharides [119]. α-glucosidase completes starch digestion and catalyzes the hydrolysis of these oligosaccharides into smaller and absorbable sugars as glucose [23]. Glucose is absorbed in the small intestine brush border and transported by the bloodstream to the tissues for its use and/or storage. Starch is not present as an isolated structure within the plant–cell matrix; it is often associated with other macronutrients, such as lipids or proteins, which can overall affect the digestion rate [120].

Several starch types can be identified according to their digestion rate: slowly digestible starch is digested in 20 to 120 min and produces slow/moderate changes in postprandial glycemia; quickly digestible starch is digested in less than 20 min and produces large changes in postprandial glycemia even comparable to that produced by simple sugars; very rapidly digestible starch is hydrolyzed in just a few minutes during the first digestion stage (i.e., mastication), and its effect on the postprandial glycemia is not yet clear [120]. Another additional starch category is resistant starch, which is not digested and passes to the digestion system with no effect on postprandial glycemia [121]. Furthermore, starch digestion may depend on food microstructure, which controls the accessibility to the substrate, the mobility of enzymes in the food bolus throughout the digestion process, the presence of substances that can alter/inhibit the action of the digestive enzymes (e.g., natural compounds as polyphenols or drugs as acarbose), and the intimate interactions of starch with other bolus components to preclude the direct contact between starch and enzymes [118]. The profile of each consumer, including their genetic background, metabolic status, or disease presence, among others, also modifies the starch digestion features. Indeed, it has been found that even particle size after mastication may affect the glycemic response, showing the complexity of this process.

**Figure 11:**
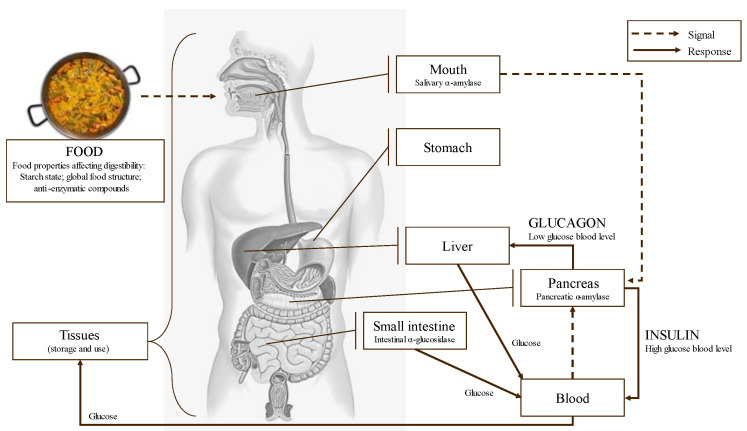
Schematic main metabolic steps and enzymes related to food digestion and glycemic response.

Foods with low glycemic responses are considered favorable to health since avoid or reduce the possibility to end up developing diabetes disease. Appropriate glycemic control is particularly important in celiac disease (Figure 11). Thus, it is mandatory to produce suitable new-generation food products to control GI responses, being the phlorotannins-enriched extracts a potential solution. Scientific evidence refutes partially the idea that a lower starch digestibility will induce a lower glycemic response [120], evidencing that glycemic response is a multifactorial phenomenon.

Polyphenols are candidates as food additives due to their health benefits. There are many publications about the potential use of polyphenols in starchy foods. For instance, Cummings and Englyst [122] studied the interactions between polyphenols from blackcurrant pomace and the main macronutrients in foods; they also examined the changes that occurred during in vitro digestion using a model that combined systems with water, wheat starch, olive oil, and/or whey protein. Wang et al. (2021) [123] studied the effects of the mixing order of tannic acid and starch on α-amylase enzyme inhibition; meanwhile, other researchers [124] evaluated corn starch’s ability to bind and carry a yerba mate polyphenolic enriched extract. Nevertheless, very few publications report the effect of the addition of seaweed and/or their nutraceuticals among starchy-based foodstuffs. Hall et al. [125] investigated the nutrient absorption of *A. nodosum* seaweed-enriched bread, and Mamat et al. [126] reported the effect of *Kappaphycus alvarezii* seaweed addition on dough’s rheological properties and the quality of bread. Along the same line, the effect of the addition of *Cladophora* spp. and *Ulva* spp. green seaweeds on the nutrient composition, caloric value, and technological and sensory evaluation of bread was reported [127]. Some recent contributions about the effect of consuming bread formulated with red seaweed *Palmaria palmata* [128]; the influence of this algae addition on the physical, antioxidant and appealing properties of bread [129] and a revision on seaweed-enriched food models for glycemic control [130] have been reported. It is expected that the number of these studies will increase in the coming years, considering the current state of the art of algal nutraceuticals and their potential healing properties.

Potential Use of Phlorotannins as Enzyme Inhibitors

As previously stated, a strategy to control the high levels of glucose in the blood is the inhibition of α-amylase and α-glucosidase, which are digestive enzymes involved in the breakdown of starch and oligosaccharides [131,132], allowing the control of postprandial glucose in diabetic patients [133,134]. In this sense, polyphenols are widely known due to their ability to associate with macromolecules, so could be applied to inhibit diverse digestive enzymes, especially α-amylase and α-glucosidase but also maltase and/or sucrase [135]. Indeed, several in vitro studies have reported the enzyme inhibitory activity of polyphenols from different sources (persimmon, sorghum, rowanberry, and almond seeds, among others) on α-amylase and α-glucosidase enzymes [136]. Along the same line, Ref. [137] showed that flavonoids inhibited glucose absorption in the intestine, a promising result for the selective inhibition of specific pathways and the development of tailor-made treatments to each patient. The ability of polyphenols to inhibit digestive enzymes is related to their well-documented interactions with some proteins and polysaccharides. Polyphenol–polysaccharide interactions are due to noncovalent (hydrogen bonds and hydrophobic interactions) or covalent interactions [138]. These interactions are largely influenced by the food matrix structure and processing conditions. 

This is a promising research line that remains partially unexplored in the case of phlorotannins, but it may be a powerful contributor to the control of the bioaccessibility, bioavailability, and antienzymatic capacities of this new generation of functional foods. Studies on the use of brown seaweed extracts or isolated phlorotannin fractions, regarding their ability to suppress carbohydrate digestion, have been conducted over the last two decades. The inhibitory capacities of polyphenols against α-amylase and α-glucosidase have been studied. For instance, researchers found that the enzymatic inhibition activity of phlorotannin-enriched extracts, reporting *A. nodosum* extracts as the strongest α-amylase inhibitors and *Fucus vesiculosus* extracts as the best α-glucosidase inhibitors [139]. However, diverse characteristics must be considered, such as extract manufacture, in vitro study conditions, the substrate used, and the harvesting period of seaweeds, among others [140]. Additionally, the data are difficult to compare because the methodologies, reagents, and origins of the assayed materials have difference. A summary of published studies of polyphenols obtained from *A. nodosum* (Table 3) and other seaweeds (Table 4) with the experimental conditions used to analyze the in vitro inhibition against α-amylase and α-glucosidases is reported.

Table 3 presents comprehensive data on the inhibitory capacities of the polyphenols derived from *A. nodosum* seaweed against α-amylase and α-glucosidase digestive enzymes. This table includes crucial information such as extraction conditions (method, number of replications, sample-to-solvent ratio, extraction time, and solvent used), inhibitor, substrate, enzyme, reaction conditions (temperature, time), and IC_50_ values. The findings strongly suggest that the polyphenols from *A. nodosum* seaweed hold significant potential as inhibitors of α-amylase and α-glucosidase enzymes. However, it is important to note that the inhibitory activity exhibits variation depending on the extraction conditions and the specific enzyme involved. Notably, the IC_50_ values reported in Table 3 range from remarkably low concentrations (e.g., 0.1 µg/mL) [70] to relatively higher concentrations (e.g., 520 µg/mL) [58]. This wide range suggests that the polyphenols from *A. nodosum* seaweed can effectively inhibit the target enzymes even at relatively low concentrations. Furthermore, the diverse IC_50_ values observed among different substrates and enzymes indicate that the inhibitory activity may be substrate- and enzyme-specific. Overall, the data presented in the third column of Table 3 underscore the potential of polyphenols from *A. nodosum* seaweed as inhibitors of α-amylase and α-glucosidase enzymes, emphasizing the critical role of extraction conditions and the inherent variability in inhibitory potency. This provides significant insights for researchers interested in exploring the potential health benefits of polyphenols derived from *A. nodosum* seaweed.

Authors have applied conventional SLE to produce bioactive extracts despite other techniques being more efficient in extracting bioactive compounds. Therefore, the application of those techniques suggests a promising and unprecedented field of study to demonstrate that seaweeds are a good option to produce antidiabetic products when they are obtained via a more-efficient and less resource-consuming extractive technique. Table 4 shows the capacities of some isolated polyphenolic compounds from seaweeds to inhibit carbohydrates digesting enzymes. It should be highlighted that in the last decade, *Ecklonia* spp. and *Eisenia* spp. have been, by far, the most commonly explored seaweed.

Table 4 presents a comprehensive analysis of the bibliographic inhibitory capacities of polyphenols from various seaweed species against α-amylase and α-glucosidase digestive enzymes, including extraction conditions, inhibitor type, substrate, enzyme, reaction conditions, and IC_50_ values for the following species: *Alaria marginata*, *Ecklonia cava*, *Ecklonia stolonifera*, *Eisenia bicyclis*, *Fucus distichus*, *Ishige foliacea*, *Ishige okamurae*, *Pyropia fallax*, *Saccharina groen-landica*, *Saccharina latissima*, *Sargassum aquifolium*, *Sargassum duplicatum*, *Sargassum filipendula*, *Sargassum polycystum*, *Sargassum ringgoldianum*, *Sargassum siliquosum*, *Triticum aestivum*, and *Ulva lactuta*. Notably, researchers have displayed a greater interest in investigating the impact of different extraction solvents, times, and temperatures rather than the extraction method itself. Previous studies have revealed that innovative methods such as UAE using water can yield superior outcomes compared with organic solvents such as acetone [41]. The inhibitors employed in the experiments summarized in Table 4 varied depending on the seaweed species, while the substrates utilized included wheat starch, p-NPG (p-nitrophenyl-α-D-glucopyranoside), potato starch, and amylopectin. The assayed enzymes were α-amylase and α-glucosidase, which play essential roles in carbohydrate digestion. Reaction conditions encompassed temperatures within a narrow range of 25 to 37 °C, with varying reaction times ranging from 5 min to 2 h. The IC_50_ values represent the concentration at which the inhibitor achieves 50% inhibition of enzyme activity, ranging from 51.6 nM reported by [148] using an extract of *Eisenia bicyclis* seaweed to 0.026 mg/mL reported by [27] using an extract of *Ecklonia stolonifera*. Seaweeds such as *Ecklonia cava* and stolonifera or *Eisenia bicyclis* exhibited notable inhibitory effects against α-amylase and α-glucosidase enzymes, with IC_50_ values from micromolar to millimolar concentrations. These observed inhibitory effects of seaweed extracts on α-amylase and α-glucosidase enzymes underscore their potential applications in managing conditions related to carbohydrate digestion and metabolism. By inhibiting these enzymes, seaweed extracts may aid in regulating blood sugar levels and could contribute to the development of functional foods or supplements for individuals with diabetes or those seeking to control their carbohydrate intake.

Apart from the potential of phlorotannins from brown seaweeds as antidiabetic molecules based on α-amylase and α-glucosidase mechanisms, there are alternative metabolic routes or strategies that can be modulated using phlorotannins. A recent study cited several examples of these routes, including the inhibition of angiotensin-converting enzymes, aldose reductase, dipeptidyl peptidase-4, and protein tyrosine phosphatase 1B enzyme [18]. Angiotensin-converting enzymes play a crucial role in regulating blood pressure by controlling the volume of fluids in the body. Inhibiting these enzymes can help manage hypertension and potentially impact glucose regulation. Aldose reductase is an enzyme involved in the metabolism of glucose. By inhibiting aldose reductase, the conversion of glucose to sorbitol can be reduced, which may be beneficial in managing diabetic complications. Dipeptidyl peptidase-4 is an enzyme that affects glucose homeostasis by acting on incretin hormones such as glucagon-like peptide-1 and gastric inhibitory peptide (GIP). The inhibition of DPP-4 can increase insulin secretion and decrease glucagon secretion, thereby improving glucose control. Protein tyrosine phosphatase 1B is a regulator of leptin and insulin signaling pathways. Inhibiting PTP1B can enhance the sensitivity of these signaling pathways, potentially leading to improved glucose homeostasis. However, it is important to note that the objective of the current review was to assess the use of phlorotannins added to gluten-free food in relation to digestive enzymes and the management of glycemic index in this specific context.

## 6. Future Perspectives

Although brown seaweed extracts are potentially beneficial for use in food systems due to their bioactive compounds, their application is limited. Antioxidant activities are attributed to phlorotannins, but the problems related to their astringency, thermal and pH stabilities, and possible presence of contaminants limit their potential applications. Encapsulating or coating polyphenols within natural or synthetic polymers can be a promising approach to ensure the stability, bioactivity, and bioavailability of phenolic compounds. However, despite these potential solutions, the development of functional ingredients from seaweed using these technologies remains a challenging task. Additionally, the high mineral content of seaweeds, together with the high iodine content, presents a significant challenge for their inclusion in foods. Thus, further research is needed to overcome these challenges and to fully develop the potential of seaweed as a functional ingredient in food systems.

Regarding stability and solubility, phlorotannins face certain limitations that can hinder their application in controlled drug delivery. While in vitro studies have provided valuable insights into the inhibitory capacities of phlorotannins, it is essential to note that their direct transferability to real food consumption scenarios is not straightforward. To address the challenges posed by factors such as oxygen, pH, ions, light, and temperature, widely used strategies involve integrating phlorotannins within matrices and developing micro- or nanostructures (nanocarriers) using nanotechnology techniques applied to food science. This integration ensures contact with digestive enzymes in the small intestine, facilitating optimal delivery and improving bioavailability and the expected health benefits associated with these innovative food formulations. Incorporating phlorotannins into food formulations enhances them and helps to mitigate the rate of polyphenol deterioration.

With the current advances in chemical and engineering technologies for the extraction and identification of bioactive compounds in seaweeds, new potential food ingredients with beneficial activities for human health and nutrition has been developed in the last years. Promising data from in vitro and animal studies have been reported; however, the effects of polyphenols on glucose homeostasis in human beings are still under discussion. Further research will determine the most biologically active polyphenols via screening and its derivatives and provide drug candidates for the pharmaceutical purposes to the reduction in or regulation of diet-linked dysfunctions. 

Optimizing and expanding the use of brown algae and their bioproducts will be the next step to exploring innovative applications in the food, medical, pharmaceutical, and/or cosmetic industries. The literature described in this review regarding extraction methods and the use of seaweed biopolymers as food additives in starchy-based systems and as digestive enzyme regulators evidences that seaweeds may be an extensive resource in the future. In conclusion, robust analytical techniques to quantify and characterize polyphenols in seaweeds is vital for industries to translate bioactive seaweed into commercial products. In the case of seaweed-starch-based systems, the presence of phenolic acids during corn starch gelatinization affects its pasting properties, particularly its thermal stability and viscosity during cooling. The addition of phenolic acids to starch-based food allows slowing and reducing the enzymatic hydrolysis of starch.

## Figures and Tables

**Table 1:** Overview of extraction conditions (method, extractant, liquid–solid ratio, time, and temperature) to obtain phlorotannins-enriched extracts from *Ascophyllum nodosum*.

Method	Extractant	LS (g_sol_/g_algae_)	Time	Temperature	TPC	Reference
SLE	Ethanol	90	30 min	30 °C	0.7 mg_PE_/mg_extract_	[47]
SLE	Water	100	1 h	65 °C	7.3 mg_PE_/g_extract_	[54]
	Ethanol (30% *v*/*v*)	5	30 min	25 °C	4.1 mg_PE_/g_extract_	
	Ethanol (80% *v*/*v*)	10	20 (+5) h	25 (+65) °C	3.4 mg_PE_/g_extract_	
SLE	Acetone (70% *v*/*v*)	20	3 h	rt	24.5 mg_PE_/g_extract._	[13]
SLE	Ethanol (50% *v*/*v*)	15	4 h	20 °C	0.2 g_PE_/g_extract_	[53]
UAE		10	30 min	NS (35 kHz)	0.4 g_PE_/g_extract_	
UAE	Ethanol (40% *v*/*v*)	50	30 min	60 °C	0.5 g_PE_/g_extract_	[46]
SLE	Water	10	24 h	4 °C	52 mg_PE_/g_extract_	[55]
	Methanol (50% *v*/*v*)				77 mg_PE_/g_extract_	
	Ethanol (75% *v*/*v*)				95.4 mg_PE_/g_extract_	
	Dioxolane (75% *v*/*v*)				90 mg_PE_/g_extract_	
	1,3-propanediol				98.5 mg_PE_/g_extract_	
UAE	Ethanol (50% *v*/*v*)	10	30 min	rt	46.6 mg/g_extract_	[56]
SLE	Methanol (70% *v*/*v*)	10	4 h	rt	0.5 g_GAE_/g_extract_	[57]
MAE (2.45 GHz)			15 min	110 °C	1.4 g_GAE_/g_extract_	
SLE	Methanol (70% *v*/*v*)	10	4 h	rt	0.5 mg_GAE_/g_d.b_	[58]
MAE (2.45 GHz)			15 min	110 °C	1.4 mg_GAE_/g_d.b_	
UAE (80 W/cm^2^)	Acetone (70% *v*/*v*)	30	4 min	<35 °C	31.8 mg_PE_/g_d.b_	[59]
SLE	Water	20	150 min	70 °C	0.17 mg_PE_/g_d.b_	[60]
	HCl (0.1 M)				0.11 mg_PE_/g_d.b_	
UAE (35.6 W/cm^2^)	Water		15 min	<35 °C	0.16 mg_PE_/g_d.b_	
	HCl (0.1 M)				0.13 mg_PE_/g_d.b_	
UAE (75.8 W/cm^2^) + SLE	HCl (0.03 M)	10	10 min + 22 h	<35 °C	135.7 mg_GAE_/g_d.b_	[61]
UAE (75.8 W/cm^2^)	HCl (0.03 M)	10	25 min	<35 °C	143.1 mg_GAE_/g_d.b_	[62]
SLE	Water	10	60 min	20 °C	178.0 mg_GAE_/mL	[63]
	HCl (5 mM)				210.0 mg_GAE_/mL	
SLE	Water	20	24 h	rt	70.5 mg_PE_/g_extract_	[43]
	Ethanol (80% *v*/*v*)	10			66.3 mg_PE_/g_extract_	
	Acetone (80% *v*/*v*)				155.9 mg_PE_/g_extract_	
PLE	Water	2	NS	120 °C (1500 psi)	93.4 mg_PE_/g_extract_	
	Ethanol (80% *v*/*v*)			100 °C (1000 psi)	101.3 mg_PE_/g_extract_	
	Acetone (80% *v*/*v*)			60 °C (1000 psi)	127.4 mg_PE_/g_extract_	
SLE	Methanol (60% *v*/*v*)	15	3 h	40 °C	4.5 mg_GAE_/g_d.b_	[64]
SLE	Ethanol (50% *v*/*v*)	12.5	90 min	80 °C	38.8 mg_PE_/g_d.b_	[65]
SLE	Water	20	24 h	rt	138.0 mg_PE_/g_extract_	[66]
	Acetone (70% *v*/*v*)				159.0 mg_PE_/g_extract_	

Note: g_algae_, grams of algae; g_d.b_, grams of algae in dry basis (d.b); g_sol_, grams of solvent MAE, microwave-assisted extraction; mg_extract_, milligrams of extract; mg_GAE_, milligrams of gallic acid equivalents; mg_PE_, milligrams of phloroglucinol equivalent; NS, not specified in the study; rt, room temperature; SLE, solid–liquid extraction; UAE, ultrasound-assisted extraction; PLE, pressurized liquid extraction; TPC, total polyphenol content determined from the extract.

**Table 2:** Main phlorotannins isolated and identified from brown seaweeds and their chemical structure.

Phlorotannin		Seaweed	Reference
2-Phloroeckol	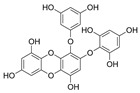	*Eisenia bicyclis*	[67]
		*Euphorbia stolonifera*	[68]
6,6′-Bieckol	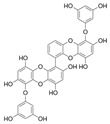	*Ishige okamurae*	[69]
7-Phloroeckol	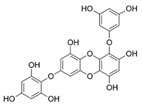	*Euphorbia stolonifera*	[68]
		*Ascophyllum nodosum*	[70]
8,8′-Bieckol	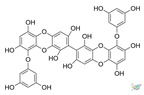	*Eisenia bicyclis*, *Ecklonia cava* and *Ecklonia kurome*	[71]
Dieckol	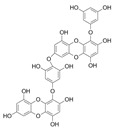	*Eisenia bicyclis*	[72]
Dioxinodehydroeckol	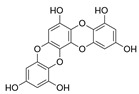	*Euphorbia stolonifera*	[68]
Diphlorethohydroxycarmalol	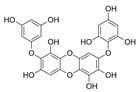	*Ishige okamurae*	[73]
Eckol	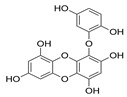	*Ecklonia kurome*	[74]
Fucodiphloroethol G	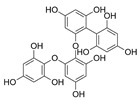	*Ecklonia cava*	[75]
Fucophlorethols	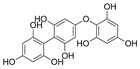	*Cystophora retroflexa*	[76]
		*Cupressus torulosa*	[77]
		*Sargassum spinuligerum*	
Phlorofucofuroeckol A	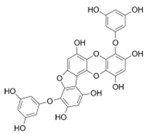	*Ecklonia kurome*	[78]
		*Euphorbia stolonifera*	[27]
Phlorofucofuroeckol B	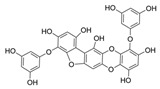	*Erica arborea*	[79]
Bifuhalol	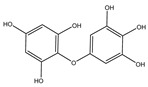	*Bifurcaria bifurcata*	[80]
Tetraphlorethols E	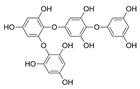	*Cystophora retroflexa*	[76]
Triphlorethol	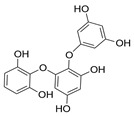	*Ecklonia cava*	[81]

**Table 3:** Inhibitory capacities against α-amylase and α-glucosidase digestive enzymes of polyphenols from *A. nodosum* seaweed.

Extraction Conditions (Method, Replicates, LS, t, Sol, T)	Inhibitor	Substrate	Enzyme	Reaction Conditions	IC_50_	Reference
SLE ×3 10 g_sol_/g_algae_ 4 h Methanol (70% *v*/*v*) rt	Raw extract	Potato starch (1% *w*/*w*)	α-amylase (1 U/mL)	t = 10 min T = 37 °C	0.052 mg/mL	[58]
		p-NPG (6 mM)	α-glucosidase (0.2 mg/mL)	t = 10 min T = 37 °C	0.52 mg/mL	
SLE 5 g_sol_/g_algae_ Overnight Chloroform rt	Raw extract	p-NPG (10 mM)	α- glucosidase (2 U/mL)	t = 10 min T = 28 °C	194.2 µg/mL	[141]
SLE 5 g_sol_/g_algae_ Overnight EtOH rt					36.3 µg/mL	
SLE 5 g_sol_/g_algae_ Overnight Acetone (70% *v*/*v*) rt					13.0 µg/mL	
SLE (x3) 40 g_sol_/g_algae_ 3 h Water 25 °C	Raw extract (10 mg/mL)	Wheat starch (1% *w*/*v*)	α-amylase (NS)	t = 10 min T = 25 °C	* 10%	[139]
		p-NPG (5 mM)	α-glucosidase (0.1 U/mL)	t = 5 min T = 37 °C	* 8%	
SLE (x3) 10 g_sol_/g_algae_ Overnight MetOH NS	Raw extract	Potato starch (1% *w*/*v*)	α-amylase (0.4 mg/mL)	t = 2 h T = 37 °C	0.1 µg/mL	[70]
		p-NPG (2 mM)	α-glucosidase (10 mg/mL)	t = 2 h T = 37 °C	19.0 µg/mL	
SLE 20 g_sol_/g_algae_ 30 min Water 80 °C	Raw extract	Wheat starch (1% *w*/*w*)	α-amylase (13 U/mL)	t = 10 min T = 25 °C	1.34 µg	[22]
		p-NPG (5 mM)	α-glucosidase (NS)	t = 20 min T = 37 °C	0.24 µg	
NS NS NS NS rt	Commercial raw extract	Corn starch (1% *w*/*w*)	α-amylase (0.2 U/mL)	t = 5 min T = 20 °C	2.8 µg/mL	[142]
		p-NPG (NS)	α-glucosidase (NS)	t = 20 min T = 37 °C	5.0 µg/mL	
SLE 12.5 g_sol_/g_algae_ 90 min EtOH (50% *v*/*v*) 80 °C	Raw extract	p-NPG (0.7 mM)	α-glucosidase (0.1 mg/mL)	t = 15 min T = 37 °C	38.0 µg/mL	[65]

g_algae_ is grams of algae; g_sol_ is grams of solvent; IC_50_ is the concentration to achieve 50% enzyme inhibition; LS is liquid–solid ratio; NS is not specified; p-NPG is p-nitrophenyl-a-D-glucopyranoside; rt is room temperature; SLE is solid–liquid extraction; Sol is solvent; t is time of extraction; T is the temperature of extraction; * results expressed concerning the control (acarbose).

**Table 4:** Inhibitory capacities against α-amylase and α-glucosidase digestive enzymes of polyphenols from seaweeds.

Seaweed spp.	Extraction (Method, Replicates, LS, t, Solvent and T)	Inhibitor	Substrate	Enzyme	Reaction Conditions (t and T)	IC_50_ % Inhibition	Reference
AM FD SG SL PF UL	SLE ×2 10g_sol_/g_algae_ 3 h MetOH (80% *v*/*v*) NS	Raw extracts (4 mg/mL)	Wheat starch 1% *w*/*v*	α-amylase NS	10 min 37 °C	AM 6.4% FD 3.4% SG 76.1% SL 75.1% PF 6.6% UL 88.0%	[143]
			p-NPG 1 mM	α-glucosidase 75 U/mg	5 min 30 °C	AM 7.9% FD 18.4% SG 65.5% SL 56.3% PF 62.2% UL 82.4%	
*Ecklonia stlonifera*	SLE x0 4g_sol_/g_algae_ 30 min Water NS	Raw extract	Wheat starch 20 mg/mL	α-glucosidase 10 U/mL	5 min 37 °C	0.026 mg/mL	[27]
*Ecklonia cava*	SLE x3 0.5g_sol_/g_algae_ 10 days MetOH rt	Fucodiphloroethol G Dieckol 6,6′-Bieckol 7-Phloroeckol Phlorofucofuroeckol A	p-NPG 3 mM	α-amylase 1 U/mL	5 min 37.5 °C	>0.5 mM	[144]
						0.1 mM	
						>0.5 mM	
						0.3 mM	
						>0.5 mM	
						>0.5 mM	
				α-glucosidase 2 U/mL	20 min 37.5 °C	19.5 µM	
						10.8 µM	
						22.2 µM	
						49.5 µM	
						19.7 µM	
*Ecklonia cava*	SLE x3 NS 10 days MetOH 80% *v*/*v* rt	Dieckol	PNPG7 3 mM	α-amylase 0.7 U	5 min 37.5 °C	0.66 mM	[145]
				α-glucosidase 1 U/mL		0.24 mM	
*Ecklonia cava*	SLE x3 50g_sol_/g_algae_ 3 h EtOH 70% *v*/*v* rt	Phlorofucofuroeckol A	PNPG7 5 mM	α-amylase 100 U	t5 min rt	6.3 µM	[146]
			p-NPG 5 mM	α-glucosidase 0.7 U		19.5 µM	
*Ecklonia stolonifera* and *Eisenia bicyclis*	SLE x3 20 g_sol_/g_algae_ 1 h MetOH 40 °C	Phloroglucinol	p-NPG 2.5 mM	α-glucosidase 0.2 U/mL	15 min 37 °C	141.2 µM	[147]
		Dioxinodehydroeckol				34.6 µM	
		Eckol				11.8 µM	
		Phlorofucofuroeckol A				1.4 µM	
		Dieckol				1.6 µM	
*Eisenia bicyclis*	SLE x3 2 g_sol_/g_algae_ 3 h MetOH rt	Phloroglucinol tetramer (1 mM)	Wheat starch 1% *w*/*v*	α-amylase NS	15 min 37 °C	96.2%	[67]
		Eckol (1 mM)				86.7%	
		Dieckol (1 mM)				76.0%	
*Eisenia bicclis*	SLE ×3 40 g_sol_/g_algae_ 3 h EtOH 25 °C	Methanolic extract n-Hexane fraction Dichloromethane fraction Ethyl acetate fraction n-Butanol fraction Water fraction Fucofuroeckol A Dioxinodehydroeckol	Potato starch 1% *w*/*v*	α-amylase 0.4 mg/mL	2 h 37.5 °C	0.5 µg/mL	[148]
						3.5 µg/mL	
						0.3 µg/mL	
						48.1 ng/mL	
						0.2 µg/mL	
						1.9 µM	
						51.6 nM	
						93.3 nM	
			p-NPG 3 mM	α- glucosidase 0.4 mg/mL	20 min 37.5 °C	>500 µg/mL	
						>500 µg/mL	
						39.98 g/mL	
						2.9 µg/mL	
						4.6 µg/mL	
						>500 µg/mL	
						42.9 µM	
						0.47 mM	
*Ishige foliacea*	SLE x3 40 g_sol_/g_algae_ 3 h MetOH 80% *v*/*v* 25 °C	Octaphlorethol A	p-NPG 5 mM	α- glucosidase 0.7 U/mL	5 min rt	0.11 mM	[149]
*Ishige okamurae*	SLE NS NS MetOH 80% *v*/*v* rt	Diphlorethohydroxycarmalol	p-NPG 5 mM	α-amylase 0.7 U	5 min rt	0.53 mM	[73]
				α-glucosidase 0.7 U/mL		0.16 mM	
*Sargassum ringgoldianum*	SLE x3 NS NS MetOH 80% *v*/*v* NS	Raw extract	PNPG7 5 mM	α-amylase 100 U	5 min rt	0.18 mg/dL	[150]
			p-NPG 5 mM	α-glucosidase 0.7 U		0.12 mg/dL	
			p-NPG 5 mM	α-glucosidase NS	20 min 37 °C	0.24 µg	
SF SA SS SP SD	SLE x3 40 g_sol_/g_algae_ 30 min EtAc 90 °C	Raw extracts	Wheat starch 1% *w*/*v*)	α-amylase 13 U/mL	10 min 25 °C	SF 27.0% SA 55.0% SS 30.0% SP 42.0% SD 36.0%	[151]
			p-NPG 5 mM	α-glucosidase 0.7 U	5 min 25 °C	SF 37.0% SA 65.0% SS 40.0% SP 52.0% SD 46.0%	
*Triticum aestivum*	SLE x2 40 g_sol_/g_algae_ 1 h EtOH 25 °C	(2-(4-(3,5-dihydroxyphenoxy)-3,5-dihydroxyphenoxy) benzene-1,3,5-triol)	Amylopectin 1% *w*/*v*	α-amylase 1 µM	5 min 37 °C	3.2 µg/mL	[152]

AM is *Alaria marginata* seaweed; EtOH is ethanol; EtAc is ethyl acetate, FD is *Fucus distichus* seaweed; g_algae_ is grams of algae; g_sol_ is grams of solvent; LS is liquid–solid ratio; MetOH is methanol; NS is not specified; PF is *Pyropia fallax* seaweed; p-NPG is p-nitrophenyl-a-D-glucopyranoside; PNPG7 is p-nitrophenyl maltoheptaoside; rt is room temperature; SA is *Sargasum aquifolium* seaweed; SD is *Sargasum duplicatum* seaweed; SF is *Sargasum filipendula* seaweed; SG is *Saccharina groenlandica* seaweed; SL is *Saccharina latissima* seaweed; SLE is solid–liquid extraction; Sol is solvent; SP is *Sargasum polycystum* seaweed; SS is *Sargasum siliquosum* seaweed; t is time; T is temperature; UL is *Ulva lactuta* seaweed; ×0 is process not repeated; ×2 is process repeated twice; ×3 is process repeated three times.

## Data Availability

Not applicable.
